# Anhedonia and Ambivalence in Schizophrenic Patients with Fronto-Cerebellar Metabolic Abnormalities: A Fluoro-D-Glucose Positron Emission Tomography Study

**DOI:** 10.4306/pi.2009.6.2.72

**Published:** 2009-06-30

**Authors:** Kyung-Min Park, Jae-Jin Kim, Jeong Ho Seok, Ji Won Chun, Hae-Jeong Park, Jong Doo Lee

**Affiliations:** 1 Department of Psychiatry and Institute of Behavioral Science in Medicine, Yonsei University College of Medicine, Seoul, Korea.; 2 Department of Nuclear Medicine, Yonsei Medical Center, Seoul, Korea.; 3 Department of Psychiatry, Hallym University Sacred Heart Hospital, Anyang, Korea.

**Keywords:** Schizophrenia, Fronto-cerebellar abnormalities, Fluoro-D-glucose positron emission tomography, Anhedonia, Ambivalence

## Abstract

**Objective:**

Prefrontal and cerebellar abnormalities have been associated with higher cognitive deficits in schizophrenia. The current study aimed to show whether or not schizophrenic patients with fronto-cerebellar functional abnormalities show more anhedonia or ambivalence.

**Methods:**

Regional cerebral metabolic activity was measured using fluoro-D-glucose positron emission tomography and was compared between 24 patients with chronic schizophrenia and 22 healthy normal volunteers. The existence of regional prefrontal hypofunction and regional cerebellar hyperfunction was investigated in each patient. Demographic and clinical variables including the emotional self-report scales were compared between the subgroups of the patients categorized according to the existence and the absence of the regional dysfunctions.

**Results:**

Comparisons between each patient and the total normal controls revealed that 14 of the total twenty-four patients had regional hypofrontal functions, whereas 11 patients had regional hypercerebellar functions. Patients with prefrontal hypofunction showed more severe anhedonia than those without prefrontal hypofunction, whereas patients with cerebellar hyperfunction compared to those without cerebellar hyperfunction had more severe ambivalence.

**Conclusion:**

It seems that fronto-cerebellar abnormalities may be associated with cardinal emotional features of schizophrenia, such as anhedonia and ambivalence.

## Introduction

Schizophrenia is a debilitating disease with heterogeneous phenomenology and diverse symptoms.^1^ Among the variety of symptoms, both anhedonia and ambivalence are considered to be fundamental in patients with schizophrenia.^2^ Anhedonia is defined as a deficit in the ability to experience pleasant emotions, and ambivalence refers to a state of simultaneous and conflicting emotional valences toward a person or object.^2^ Therefore, the two symptoms could reflect some aspects of emotional disturbances that accompany schizophrenia.

However, patients with schizophrenia have a deficit in the ability to experience positive emotions related to future activities (anticipatory pleasure) but experience normal levels of positive emotions when directly engaged in pleasant activities (consummatory pleasure).^3^ This indicates that anhedonia may be caused by the disturbances in higher cognitive functioning, e.g. executive functions such as anticipation as well as emotional processing. In addition, ambivalence essentially induces approach-avoidance conflicts and thus higher cognitive functions are required to resolve conflicting emotional valences.^4^ Emotional disturbances in individuals with schizophrenia are thought to derive from abnormal interactions between the mesolimbic system and brain regions such as the prefrontal cortex and cerebellum that are responsible for higher cognitive functions.^5^,^6^ Therefore, the psychological and neural underpinnings of these two fundamental symptoms could be based on the interface between the neural substrates of emotion and higher cognition.

The prefrontal cortex and the cerebellum in the human are two brain regions that are massively larger by approximately one-third when compared to primates that lack capacities for higher cognitive functions.^7^ In addition, tract-tracing studies show that the cerebellum has strong connectivity between the prefrontal regions, which supports the notion that the cerebellum participates in neural circuits that perform higher cognitive functions of the prefrontal cortex.^8^ Therefore, the prefrontal cortex and the cerebellum are among a variety of brain regions considered to be key substrates for higher cognitive functions.^6^,^9^

Abnormalities in higher cognitive functions, hallmarks of schizophrenia, have been consistently connected to disrupted functioning of the prefrontal cortex and the cerebellum.^9^-^12^ Thus, the aim of this study was to examine whether patients with dysfunctional activity in these brain regions would show more severe anhedonia or ambivalence than subjects with normal activity at the same loci.

Abnormalities in higher cognitive functions in schizophrenia are associated with prefrontal hypofunction rather than hyperfunction, and cerebellar hypofunction^10^,^13^ or hyperfunction.^9^,^14^ The crucial roles of the cerebellum in the circuit responsible for higher cognitive functions are coordinating or modulating the activities in the prefrontal cortex, and therefore cerebellar activities could vary across features of required higher cognitive functions. Moreover, it is very difficult to connect a regional abnormality pattern to a specific clinical symptom in each individual patient, as most imaging results in schizophrenia have been obtained from analyzing the between-group differences or the within-group correlations. This is in part due to diverse functional patterns among individual patients with the illness as well as subtle variations in functional activities in the individuals.

Therefore, we investigated abnormal regional brain functions using 2-deoxy-2-18F-fluoro-glucose-positron emission tomography (^18^F-FDG-PET) in each individual patient when compared with total normal controls in order to predict those who would have abnormal functions in the prefrontal cortex and the cerebellum. We then compared the severities of anhedonia and ambivalence between patients with and without prefrontal hypofunction, and between patients with and without cerebellar hyperfunction. We hypothesized that the severities of anhedonia and ambivalence would be significantly different between the patient subgroups.

## Methods

### Subjects

The subjects were 24 patients with schizophrenia and 22 age-matched normal, healthy volunteers. The patients were recruited from the outpatient clinics at the Severance Mental Health Hospital (SMH), and had been in follow-up for more than two years. They fulfilled the DSM-IV criteria for schizophrenia, as diagnosed using the Structured Clinical Interview for DSM-IV (SCID).^15^ The exclusion criteria for the study were any lifetime history of neurological or other significant medical illnesses and any past history of substance abuse. The normal, healthy volunteers were recruited from the local community, and were screened for any current or lifetime history of a DSM-IV Axis I disorder using the SCID. The study was carried out under the guidelines established by the institutional review board of the SMH for research involving human subjects, and written informed consent was obtained from all subjects before the study began.

Each group contained the same number of men and women, and all participants were right-handed. The mean ages of the patient and normal control groups were 30.5 years (SD=3.5, range=23-36) and 30.3 years (SD=3.1, range=25-35), respectively. The mean years of education were 12.8 (SD=2.0) and 13.0 years (SD=1.6), respectively. Age and educational background were not significantly different between the groups. At the time of positron emission tomography (PET) scanning, the mean duration of illness in the patient group was 8.4 years (SD=3.8). All patients received either one or two neuroleptics, including risperidone, olanzapine, clozapine, aripiprazole and haloperidol, with a mean chlorpromazine- equivalent dose^16^ of 586.4 mg (SD=349.5).

### Measurement of symptom severity and emotional characteristics

Symptom severity of the patients was assessed using the Positive and Negative Syndrome Scale^17^ within the week before PET scanning. The mean scores of schizophrenic symptom severities were calculated in the patient group: positive (mean=16.1, SD=3.2), negative (mean=17.0, SD=2.4), general (mean=34.0, SD=4.9), and total (mean=67.1, SD=8.9) symptoms.

Immediately after PET scanning, all subjects reported their own emotional tendencies by filling out four different self-report scales such as the physical and social anhedonia scales,^18^ the schizotypal ambivalence scale^19^ and the emotional expressivity scale.^20^ The physical anhedonia scale consists of 61 Yes-No questions and measures a deficit in the ability to experience pleasure, whereas the social anhedonia scale containing 40 items measures social withdrawal, a lack of interest and pleasure in social and interpersonal relationships. Scores of the physical anhedonia scale in the patients (mean=21.3, SD=9.6) were higher than those in the normal controls (mean=14.5, SD=6.9)(t=2.74, p<0.01), and scores of the social anhedonia scale in the patients (mean=15.5, SD=6.2) were also higher than those in the normal controls (mean=9.2, SD=5.1)(t=3.77, p<0.001). The schizotypal ambivalence scale consists of 19 Yes-No questions to identify ambivalence as characteristic of schizotypy and schizophrenia. Scores of the schizotypal ambivalence scale in the patients with schizophrenia (mean=10.6, SD=6.3) were higher than those in the normal controls (mean=6.2, SD=3.7)(t=2.95, p<0.01). The emotional expressivity scale, a 17-item 6-point scale, assesses the extent to which people outwardly display their emotions. Unlike other scales, scores of the emotional expressivity scale in the patients (mean=60.6, SD=8.6) were not significantly different from those in the normal controls (mean=65.6, SD=10.7). The demographic and scales data were analyzed using the Statistical Package for the Social Sciences (SPSS)(Version 13.0, Chicago, IL, USA) and the significance was set at p<0.05 (two-tailed).

### Positron emission tomography procedures and imaging data preprocessing

PET images were acquired using a GE ADVANCE PET scanner (GE, Milwaukee, Wisconsin, USA) which had an intrinsic resolution of 4.8 mm full width at half maximum (FWHM) and a slice thickness of 4.25 mm for a longitudinal field of view of 15.2 cm. Subjects were asked to lie still with their eyes closed in a quiet, dimly lit room for 40 minutes after receiving an intravenous injection of approximately 185 MBq (5 mCi) of ^18^F-FDG. Afterwards, an 8-min transmission scan was performed using Ge-68 rod sources to correct for attenuation. The emission scan continued in 3-dimensional mode for 15 minutes. Gathered data were then reconstructed in a 128×128×35 matrix with a pixel size of 1.95×1.95×4.25 mm using a 3-dimensional filtered back-projection algorithm employing a transaxial 8.5 mm Hanning Filter and an 8.5 mm axial Ramp Filter.^21^

Spatial pre-processing and statistical analyses were performed using Statistical Parametric Mapping (SPM2, Institute of Neurology, University College of London, UK). All reconstructed images were spatially normalized into the MNI (Montreal Neurological Institute, McGill University, CA) standard PET template to remove the inter-subject anatomical variability. An affine transformation was performed to determine the 12 optimal parameters in order to register the brain on the template. Subtle differences between the transformed image and the template were removed by the nonlinear registration method using the weighted sum of the pre-defined smooth basis functions involved in discrete cosine transformation. Spatially normalized images were smoothed by convolution with an isotropic Gaussian kernel with 14 mm FWHM to increase the signal-to-noise ratio and accommodate for variations in subtle anatomical structures.

### Statistical analysis

The effects of global metabolism were removed by normalizing the count of each voxel to the mean count of the brain (proportional scaling in SPM). In order to demonstrate abnormalities in each case, comparisons were made between each patient and the 22 total normal controls. Significant decreases and increases of the adjusted regional cerebral metabolism were obtained using t-statistics at every voxel. To facilitate interpretation, the t-values were transformed to Z-scores in the standard Gaussian distribution. The clusters consisting of a minimum of 50 contiguous voxels with the threshold of an uncorrected p<0.001 were considered to be significant. According to the a priori hypothesis that there would be prefrontal hypofunction and cerebellar hyperfunction in patients with schizophrenia, the existence or the absence of regional abnormal clusters in the prefrontal cortex and in the cerebellum were categorized into subgroups using the Talaraich coordinate system.^22^ Thereafter, demographic and clinical variables were compared between these defined subgroups using χ^2^-tests for categorical variables or independent t-tests for continuous variables. We used the Statistical Package for the Social Sciences (SPSS) (Version 13.0, Chicago, IL, USA), and significance of the differences was accepted when p<0.05 (two-tailed) in the analyses.

## Results

As demonstrated in Table 1, comparisons between each patient and the total normal controls revealed that 14 of the total twenty-four patients had regional hypofrontal functions, and 11 patients had regional hypercerebellar functions. Six patients had both regional hypofrontal and hypercerebellar functions, 8 patients had only regional hypofrontal functions, and 5 patients had only regional hypercerebellar functions. However, 5 patients had no abnormalities in these brain regions.

**FIGURE 1:**
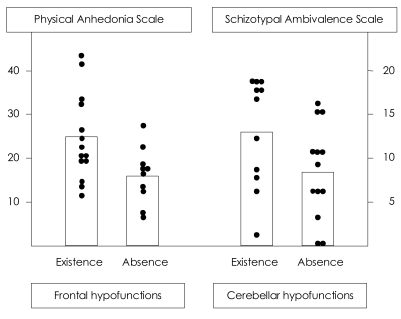
Differences of the physical anhedonia scale scores and schizotypal ambivalence scale scores between the existence and the absence of the regional hypofrontal and hypercerebellar functions.

Table 2 shows subgroup means of demographic and clinical variables according to whether or not the regional metabolic abnormalities were apparent. Most variables were unchanged whether or not abnormal functioning was evident. As shown in Figure 1, the only difference between the patient subgroups with and without regional hypofrontal function was observed in the physical anhedonia scale. On the other hand, the only difference between the patient subgroups with and without regional hypercerebellar function was identified in the schizotypal ambivalence scale.

## Discussion

Our unique approach, in which each case and the total normal controls were compared, demonstrated that only 6 patients (25.0%) had both regional hypofrontal and hypercerebellar functions, whereas 5 patients (20.8%) had no abnormalities in these brain regions. It is well known that schizophrenia is a heterogeneous disorder. Previous studies have reported inconsistent brain activities in patients with schizophrenia during the resting state and even during the task-stimulating condition.^23^,^24^ As described in Table 1, regional activity patterns are variable among the patients, suggesting that results from the group comparison could be dependent on the composition of subjects. Therefore, the abnormalities in the two brain regions could not be generalized to problems of all patients with schizophrenia, but rather associated with some critical features rather than common problems in the patients with schizophrenia.

The patients with prefrontal hypofunction compared to those without prefrontal hypofunction showed more severe physical anhedonia, whereas the patients with cerebellar hyperfunction relative to those without cerebellar hyperfunction exhibited more severe ambivalence (Table 2, Figure 1). These results could suggest that the two symptoms have different neural correlates within the brain such as prefrontal-thalamic-cerebellar circuitry.^25^

In fact, patients with schizophrenia had a normal internal experience of positive emotion in the moment (consummatory pleasure), but they failed to anticipate or expect that future events would elicit these emotions (anticipatory pleasure).^3^ Behavioral studies indicated deficits in reward-related decision-making in patients with schizophrenia,^26^ and neuroimaging studies suggested that they failed to recruit reward-related brain regions while predicting future reward.^27^-^30^ Moreover, a recent study^31^ showed that they were impaired in amplification but not suppression of pleasant expressions. These results could suggest that hypofunction of the brain regions responsible for the anticipation of future pleasant activities is a critical neural substrate of anhedonia. It is well-established that the prefrontal cortex among various brain areas plays key roles in anticipation;^32^ in particular, the prefrontal cortex may be preferentially engaged in the positive emotions.^33^ Furthermore, the hypofrontality of schizophrenia is associated with impairments in executive functions such as anticipation.^32^ These findings our consistent with our results that patients with prefrontal hypofunction have more severe anhedonia than those without prefrontal hypofunction.

On the other hand, core roles of the cerebellum in the higher cognitive functions include rapid, online error detection, modulation, and coordination, culminating in the production of mental representations in a fine-tuned pattern. The cerebellum is activated in a variety of mental activities including facial recognition, emotional attribution, theory of mind, directed attention and many types of memory.^6^ In order to resolve ambivalence by simultaneously representing conflicting emotional valences,^4^ individuals may have to detect the errors driven from the conflicting emotional representations, modulate and coordinate the representations and the errors in a rapid and online manner, and then produce the integrated mental representations in a fine-tuned pattern. Therefore, it may be possible that hyperfunction of the cerebellum may reflect enhanced difficulty in resolution of ambivalence. This possibility could be supported by our finding indicating that the patients with cerebellar hyperfunction have more severe ambivalence than those without cerebellar hyperfunction.

A limitation of our study was that the patients with schizophrenia were all medicated with antipsychotics. There are reports that antipsychotic medications affect cerebellar and even prefrontal functions.^34^,^35^ However, it has to be emphasized that both prefrontal hypofunction and cerebellar hyperfunction were found in drug-naїve first-episode patients with schizophrenia.^14^ Another caveat is that we used self-report scales to measure the emotional tendencies. Because of the problem of low reliability in the scales, it is possible that the exact emotional states of the subjects were not reflected. Another limitation is that our major findings were obtained from the comparisons between patient subgroups, and there were only a small number of subjects in each patient subgroup. In addition, the validity of our approach, in which each case and the total normal controls were compared, might need to be confirmed by future investigations, though it was used only to set up the patient subgroups.

In conclusion, this study found that the patients with prefrontal hypofunction tended to have more severe anhedonia than those without prefrontal hypofunction, whereas the patients with cerebellar hyperfunction tended to show more severe ambivalence than those without cerebellar hyperfunction. Therefore, these results support the notion that prefronto-cerebellar abnormalities are associated with cardinal features of schizophrenia, such as anhedonia and ambivalence.

## Figures and Tables

**TABLE 1:**
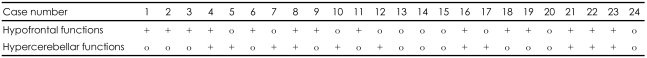
Existence or absence of regional hypofrontal and hypercerebellar functions in an individual patient with schizophrenia when compared with the normal comparison group

+: existence, o: absence.

**TABLE 2:**
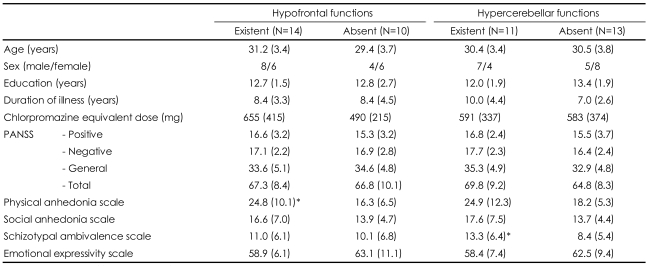
Subgroup means (SD) of demographic and clinical variables

^*^p<0.05. PANSS: positive and negative syndrome scale
